# Is Systemic Inflammatory Response Index (SIRI) a Reliable Tool for Prognosis of Gastric Cancer Patients Without Neoadjuvant Therapy?

**DOI:** 10.7759/cureus.36597

**Published:** 2023-03-23

**Authors:** Hilmi Yazici, Sevket Cumhur Yegen

**Affiliations:** 1 General Surgery, Marmara University Pendik Training and Research Hospital, Istanbul, TUR

**Keywords:** systemic inflammation response index, neoadjuvant chemoradiotherapy, survival, overall, gastric cancer

## Abstract

Background: The systemic inflammatory response index (SIRI), which depends on peripheral neutrophil, monocyte, and lymphocyte count, was found as an effective prognostic indicator for various malignancies. This study aims to investigate the predictive value of preoperative SIRI in the prognosis of gastric cancer patients without neoadjuvant therapy.

Methods: The patients who underwent gastric cancer surgery in Marmara University Hospital's General Surgery Department between 2019 and 2021 were analyzed retrospectively. SIRI was calculated from preoperative peripheral blood samples’ neutrophil, lymphocyte, and monocyte count. The optimal cut-off value for SIRI was calculated by the receiver operating characteristics (ROC) curve and was found to be 1.35. The clinicopathological outcomes and overall survival (OS) were analyzed under two groups according to the SIRI values lower or higher than 1.35.

Results: The number of eligible patients was 199. The median follow-up time was 25 (1-56) months. The higher SIRI was associated with male gender (p = 0.044), lower serum albumin (0.002) level, and Clavien-Dindo (CD) Grade III and higher complications (p = 0.018). However, there was no significant difference between the groups regarded pathological tumor, nodes, and metastases (TNM) stages, histological grade, and Lauren Type. In addition, OS and stage-specific OS were similar between the groups.

Conclusions: SIRI may be a valuable and effective predictive indicator for postoperative morbidity. The prognostic performance of SIRI on long-term OS is still controversial. Further investigations are needed into this topic.

## Introduction

Gastric cancer is one of the most common cancers worldwide. According to the latest global data, it is the sixth most common cancer and the third leading cause of death [1]. Result of this, it remains a challenging health problem for all clinicians. Extended surgical resection with a proper lymph node dissection is still the only curative treatment for gastric cancer [2]. Despite many advances in surgical techniques and chemotherapeutic drugs, gastric cancer still has a poor prognosis. According to the latest data, five-year overall survival (OS) rates are still below 30%, and the median survival for advanced gastric cancer is almost less than one year [3].

Systemic inflammation has been identified as an important factor in tumor carcinogenesis, development, and prognosis [4]. In addition, systemic inflammatory cells were shown to have a critical role in systemic inflammation and were thought to have a role in tumor prognosis [5]. The relationship between peripheral blood cells and cancer prognosis has started to be evaluated in more depth. Therefore, many studies have been conducted showing the relationship between neutrophil to lymphocyte ratio (NLR), monocyte to lymphocyte Ratio (MLR), and platelet to lymphocyte ratio (PLR) and the prognosis of various cancers [6-9]. Neoadjuvant chemo/radiotherapy (NACT) has been reported to take a more critical role in gastric cancer management in recent years [10-11]. However, recent studies showed that chemotherapy could suppress the systemic inflammatory response and affect inflammatory cells [12-13].

In recent years, the systemic inflammatory response index (SIRI), based on the peripheral blood neutrophil, monocyte, and lymphocyte, has been argued as a new prognostic index for many various cancers [14-15]. However, only a few studies were designed to evaluate the prognostic value of SIRI on gastric cancer patients without NACT.

This study aims to investigate the predictive performance of perioperative outcomes and the prognostic significance of preoperative SIRI in patients who underwent gastric cancer surgery without receiving NACT.

## Materials and methods

Data regarding patients who underwent gastric cancer surgery in Marmara University Hospital's General Surgery Department between January 2019 and December 2021 were retrospectively analyzed. The patients with gastric adenocarcinoma and who underwent surgery were included in the study. As a result of affecting systemic inflammation, patients who received NACT were not included in the study. The patients diagnosed with other than adenocarcinoma, and with an unresectable or metastatic disease were excluded. Operation procedures were performed according to the Japanese Gastric Cancer Treatment Guidelines 5th English Edition [2]. Postoperative complications were evaluated according to the CD classification, and grade III and higher complications described as requiring surgical, endoscopic, or radiological intervention were included in the analysis [16]. Pathological outcomes of the patients were evaluated according to *The Eighth Edition AJCC Cancer Staging Manual *[17].

Patient demographics, preoperative laboratory results, Grade III and higher complications, pathological outcomes, and follow-up records were obtained from hospital's computer database. SIRI was calculated with SIRI = neutrophil count × monocyte count/lymphocyte count formula. The optimal cut-off value for SIRI was calculated by receiver operating characteristics (ROC) curve analysis. The choice of threshold based on the Youden index (sensitivity + specificity − 1) was used to estimate sensitivity and specificity. The cut-off value for SIRI was found to be 1.35. Therefore, patients were examined under two groups: SIRI ≤ 1.35 and SIRI > 1.35. The patients were followed up according to the relevant guidelines [18].

This study was approved by the Ethics Committee of the Hospital (numbered 03.02.2023.228) in accordance with the guidelines of the Declaration of Helsinki, and informed consent was obtained from all patients. The primary outcome of this study was to investigate SIRI as a predictor on the postoperative outcomes of patients underwent gastric cancer surgery. The secondary outcome of this study was to compare the two groups’ OS and stage-specific survival analysis.

Statistical analysis

The SPSS version 24.0 (SPSS Inc. IBM, Chicago, USA) was used for statistical analysis. The proportion or frequency was compared between the two groups using Fisher’s exact test or the c2 test, and differences in continuous variables were evaluated using the Student’s t-test and the Mann-Whitney U test for non-parametric values. Survival curves were estimated using the Kaplan-Meier method and compared using the log-rank test.

## Results

Between January 2019 and December 2021, 273 patients underwent gastric cancer surgery. Among them, 67 patients who received NACT were excluded. The patients who underwent noncurative resections (7) were also excluded from the study. A total number of 199 patients were included. The median age of the entire cohort was 64 (26-90). There were 130 (65%) males and 69 (35%) females.

The cut-off value for SIRI determined by the ROC curve analysis was 1.35. The patients were evaluated under two groups according to this: SIRI ≤ 1.35 and SIRI > 1.35. There were 93 patients in the lower SIRI group and 106 patients in the higher group. The median age, BMI (body mass index), operation types, postoperative hospital stay, and tumor markers were similar across the two groups (Table 1). The higher SIRI levels were related to the male gender (p = 0.044). As expected, NLR, MLR, and PLR were significantly higher in the SIRI > 1.35 group. The median albumin level was lower in the SIRI > 1.35 group, and the difference was significant (p = 0.002).

**Table 1:** Patient demographics and operative results in both groups. DSG, distal subtotal gastrectomy; PSG, proximal subtotal gastrectomy; TG, total gastrectomy; NLR, neutrophil to lymphocyte ratio; MLR, monocyte to lymphocyte ratio; PLR, platelet to lymphocyte ratio; SD, standard deviation; IQR, interquartile range; BMI, body mass index; CEA, carcinoembryonic antigen; CA, cancer antigen

Total N = 199; Median (IQR)-Mean (±SE)	SIRI ≤ 1.35 N = 93	SIRI > 1.35 N = 106	p
Age (Median)	63.5(21.1)	64.5(18.7)	0.421
Gender			0.044
Female	39	30	
Male	54	76	
BMI (Mean)	25.7(0.48)	24.5(0.39)	0.072
Surgery type			0,226
DSG	49	49	
PSG	2	7	
TG	42	50	
Postoperative hospital time (days)	5(2)	5(2)	0,164
CEA (µg/L)	2.2(2.5)	2.3(2.6)	0,941
CA 19-9 (U/mL)	4.1(7.7)	17.2(40.1)	0,066
CA 125 (U/mL)	8.7(7.8)	8.3(9.2)	0,743
Pre-operative NLR	2.1(0.7)	4.5(3.1)	<0.001
Pre-operative MLR	0.2(0.08)	0.4(0.2)	<0.001
Pre-operative PLR	122.5(95.8)	190(98.9)	<0.001
Pre-operative albumin (g/L)	4.1(0.3)	3,6(0.9)	0,002

The pathological outcomes and complications among the groups are summarized in Table 2. SIRI had no significant correlation with TNM stage, lymphovascular invasion, histological grade, and Lauren Classification. However, Postoperative CD Grade III and higher complications were significantly higher in the SIRI > 1.35 group [respectively, n = 12 (15%) vs. n: 28 (22%), p = 0.018].

**Table 2:** Postoperative outcomes in both groups. *According to Clavian-Dindo (CD) Classification SD, standard deviation; IQR, interquartile range

Total N = 199 Median (IQR)-Mean (±SE)	SIRI ≤ 1.35 N = 93	SIRI > 1.35 N = 106	p
Stage T			0.185
T1	14	9	
T2	6	6	
T3	27	23	
T4	46	68	
Stage N			0.648
N0	26	27	
N1	15	13	
N2	18	20	
N3	34	46	
Pathological Stage			0,481
Stage I	19	13	
Stage II	15	18	
Stage III	55	70	
Stage IV	4	5	
Lymphovascular Invasion			0.547
Yes	76	90	
No	17	16	
Lauren Classification			0.069
Intestinal	51	46	
Diffuse	26	28	
Mixed	16	32	
Grade			0.526
Well	23	23	
Poor	70	83	
Complication Grades*	12 (15%)	28 (22%)	0.018
≥ IIIA			
IIIA	6	17	
IIIB	3	9	
IVA	1	0	
IVB	1	0	
V	1	2	

**Figure 1:**
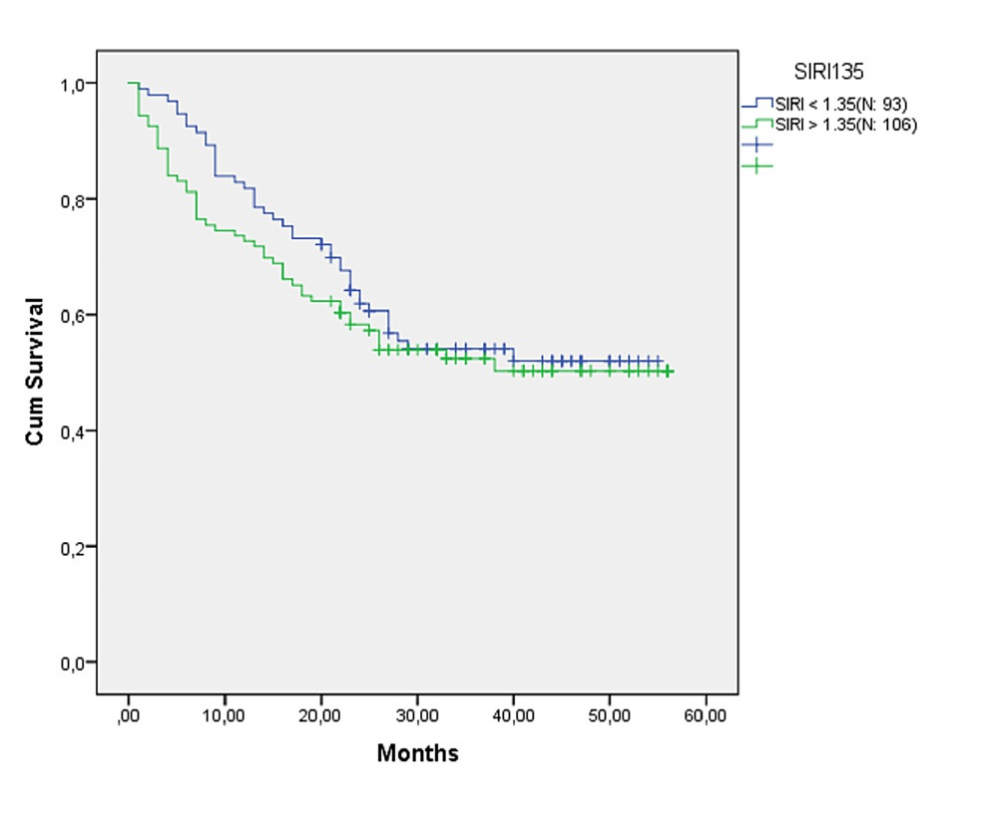
Survival analysis of both groups. Log Rank (Mantel-Cox): 0.464.

**Figure 2:**
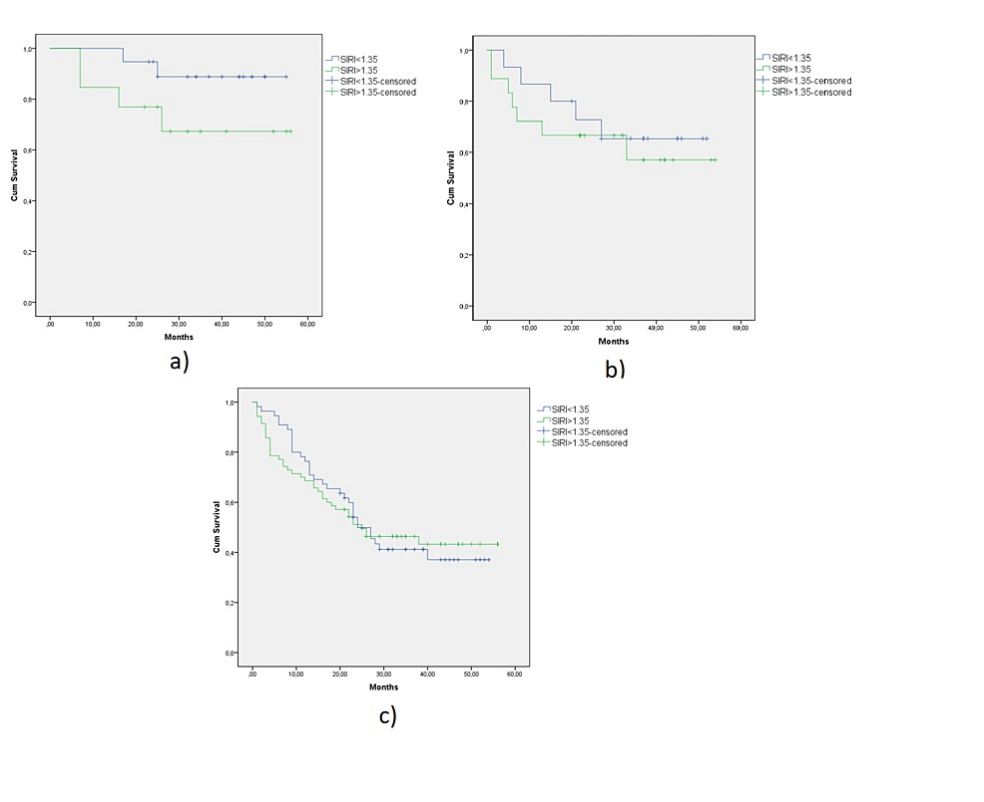
Effect of the SIRI on the survival of adenocarcinoma of the stomach patients in stage I (a), stage II (b), and stage III (c). SIRI, systemic inflammatory response index

The median follow-up of the entire cohort was 25 (1-56) months. Kaplan-Meier analysis showed that there was no statistically significant difference in OS between the two groups (Log Rank: 0.464). The survival curves of the two groups are shown in Figure 1. OS analysis according to the pathological stages was summarized in Figure 2. No significant differences were obtained between the two groups.

## Discussion

In recent years, researchers have focused more on tumor biology and behavior. Especially in the last decade, studies have shown that increased systemic inflammation may be associated with tumor prognosis [19]. Inflammatory cells can affect the tumor's microenvironment, promoting tumorigenesis and increasing tumor cell proliferation, migration, and immune escape [4]. Neutrophils, lymphocytes, and thrombocytes can reflect the immune and inflammatory processes of patients with malignancies [20]. As a result, various prognostic indexes depending on these inflammatory cells have been developing. A verified, strong tumor-specific predictor could be useful in clinical practice. Consequently, the SIRI index as one of them needs to be validated by further studies.

Previous studies showed that SIRI has a higher predictive value than NLR, MLR, and PLR. Chen et al. reported that SIRI has larger area under the curve (AUC) values than NLR, MLR, and PLR in both three years and five years follow-up in advanced esophagogastric junction cancer patients [21]. In a large-scale cohort, Li et al. reported that SIRI was significantly associated with disease-free survival and disease-specific survival in operated gastric cancer patients in both univariate and multivariate analyses [22]. However, the multivariate analysis could not verify the prognostic significance of NLR and MLR. Increased SIRI in postoperative period showed worse OS rates than those not increased [23]. In the current study, there was no significant difference in OS rates between the higher SIRI group and the lower SIRI group. A couple of reasons might explain this. First, all these studies were from eastern countries. It is well known that tumor behaviors, characteristics, and prognosis of gastric cancers can vary in different populations. In this study, according to the Lauren Classification, mixed and diffuse types were more common than in these studies. Moreover, a poor histological grade was observed in a bigger proportion of the patients. Therefore, further investigations are needed into this topic in various populations.

Chen et al. used SIRI as a prognostic indicator in gastric cancer patients receiving NACT [20]. Their cohort observed similar rates of Lauren diffuse-type carcinoma and poor histological grade. Nevertheless, as mentioned before, chemotherapy might affect immune and inflammatory responses. In our study, based on this theory, patients underwent gastric cancer surgery without NACT were analyzed. As a result, the effect of NACT on the prediction of SIRI remains unclear.

Many studies showed that systemic inflammation is strongly related to postoperative morbidity [24-25]. Although the results of OS were not significant, severe complications were significantly higher in the SIRI > 1.35 group. Li et al. reported that postoperative complications were an independent risk factor for poor prognosis in long-term OS [26]. The median follow-up of this study was 25 months. Furthermore, results may change in more extended follow-up periods.

There are some limitations for this study. Firstly, it is the retrospective design. This is a study from a single center, and these may lead to some selection and analytical biases. Secondly, the optimal cut-off values of the prognostic indexes are different in the literature. This might affect the results in diverse patient populations. Finally, adjuvant treatment data was very heterogenous; hence, it was excluded from the study.

## Conclusions

Prognostic indicators such as SIRI could be helpful in the management of malignant diseases. It showed satisfying results on the prediction of postoperative complications. However, its performance on the prognosis of gastric cancer is still controversial. Finally, further prospective, randomized-controlled studies are needed on this topic.
